# Defined plant extracts can protect human cells against combined xenobiotic effects

**DOI:** 10.1186/1745-6673-6-3

**Published:** 2011-01-20

**Authors:** Céline Gasnier, Claire Laurant, Cécile Decroix-Laporte, Robin Mesnage, Emilie Clair, Carine Travert, Gilles-Eric Séralini

**Affiliations:** 1 Laboratory of Biochemistry EA2608, Institute of Biology, University of Caen, France; 2 CRIIGEN and Risk Pôle MRSH, CNRS, University of Caen 14032, France; 3 Sevene Pharma, 30170 Monoblet, France

## Abstract

**Background:**

Pollutants representative of common environmental contaminants induce intracellular toxicity in human cells, which is generally amplified in combinations. We wanted to test the common pathways of intoxication and detoxification in human embryonic and liver cell lines. We used various pollutants such as Roundup residues, Bisphenol-A and Atrazine, and five precise medicinal plant extracts called Circ1, Dig1, Dig2, Sp1, and Uro1 in order to understand whether specific molecular actions took place or not.

**Methods:**

Kidney and liver are major detoxification organs. We have studied embryonic kidney and hepatic human cell lines E293 and HepG2. The intoxication was induced on the one hand by a formulation of one of the most common herbicides worldwide, Roundup 450 GT+ (glyphosate and specific adjuvants), and on the other hand by a mixture of Bisphenol-A and Atrazine, all found in surface waters, feed and food. The prevention and curative effects of plant extracts were also measured on mitochondrial succinate dehydrogenase activity, on the entry of radiolabelled glyphosate (in Roundup) in cells, and on cytochromes P450 1A2 and 3A4 as well as glutathione-S-transferase.

**Results:**

Clear toxicities of pollutants were observed on both cell lines at very low sub-agricultural dilutions. The prevention of such phenomena took place within 48 h with the plant extracts tested, with success rates ranging between 25-34% for the E293 intoxicated by Roundup, and surprisingly up to 71% for the HepG2. By contrast, after intoxication, no plant extract was capable of restoring E293 viability within 48 h, however, two medicinal plant combinations did restore the Bisphenol-A/Atrazine intoxicated HepG2 up to 24-28%. The analysis of underlying mechanisms revealed that plant extracts were not capable of preventing radiolabelled glyphosate from entering cells; however Dig2 did restore the CYP1A2 activity disrupted by Roundup, and had only a mild preventive effect on the CYP3A4, and no effect on the glutathione S-transferase.

**Conclusions:**

Environmental pollutants have intracellular effects that can be prevented, or cured in part, by precise medicinal plant extracts in two human cell lines. This appears to be mediated at least in part by the cytochromes P450 modulation.

## Background

Biochemical activities are generally detailed per compound in cellular research, although human cells are exposed daily to mixtures of xenobiotics and plant compounds. However, medicinal extracts may be claimed to prevent or cure chemical intoxications, but few of these are tested for their mechanisms of actions or cellular impacts. With a view to tackle this issue, we have first characterized the mechanisms of intoxication of two human cell lines with mixtures of common environmental pollutants. One of them is Roundup (R), the most widely used herbicide worldwide, the residues of which are common in surface waters [1]. These residues also enter the food chain [2], even through genetically modified edible plants [3]. R is made up from a mixture of an isopropylamine salt of glyphosate (G), quantitatively a minor compound, and various specific adjuvants depending on the formulation [4]. We have previously characterized some toxic effects and their pathways for several R formulations, and endocrine disrupting actions at nontoxic levels. This was proved with human cell lines JEG3, E293, HepG2, Hep3B, and fresh umbilical cord or placental cells [5-9]. The second xenobiotic mixture of Bisphenol-A (BPA) and Atrazine (Az) is from products commonly found in the environment; BPA, a plastic compound found in the food chain, and Atrazine a major herbicide with its derivatives (also in surface waters) the toxicity of which we have studied alone [10]. In the present study, we chose the human embryonic kidney cell line E293, for the intoxication/detoxification models, because it represents a very sensitive model, and then HepG2, as it is one of the most well known and available cell lines derived from the human liver, which is the major detoxification organ on a par with the kidneys. Moreover HepG2 cells are characterized for xenobiotic metabolism enzymes, mainly cytochromes P450 CYP1A2, CYP3A4, and glutathione S-transferase (GST), [11-13] measured in this work.

Detoxifying mechanisms are frequently claimed to be enhanced by plant extracts [14,15]. We have tested the ability of 5 newly characterized drugs, Circ1, Dig1, Dig2, Sp1 and Uro1 to protect or cure human cells before or after intoxication. The composition of each drug was previously developed by Sevene Pharma Company and is represented in Table 1. According to the scientific literature, the protective properties of the plants involved are very large. For instance, some herbal extracts of Circ1 can be hepatoprotective [16,17]. Some Sp1 compounds not only feature anti-mutagenic activities, but also provide a protection against oxidative stress, as well as anti-tumor and anti-inflammatory effects [18-20]. Among others, the herbal extracts of Uro1 have anti-inflammatory, anti-oxidative and anti-microbial activities [21-23]. However, the combined effects of the new drugs have never been tested at the cellular level. We focused here on Dig1 and particularly Dig2 for the potential of digestive detoxification or hepato-protective synergistic effects provided by some of their compounds [24-29]. It was therefore quite interesting to compare these general findings on plant extracts to some biochemically precise markers that could be modified in human hepatocytes, such as cytochromes P450, glutathione S-transferase (GST), and first of all in cellular viability studies on mitochondrial succinate dehydrogenase (SD). This could enable us to understand the action pathways of these mixtures used as medicinal plants *in vivo*.

**Table 1:** Plant composition of preventive and/or curative products.

Products	Plant extracts
	*Taraxacum officinalis*
**Dig1**	*Arctium lappa*
	*Berberis vulgaris*

	*Chelidonium majus*
**Dig2**	*Rhamnus frangula*
	*Raphanus sativus*

	*Carduus marianus*
**Circ1**	*Pulsatilla vulgaris*
	*Berberis vulgaris*

	*Pulsatilla vulgaris*
**Sp1**	*Sambucus nigra*
	*Rumex crispus*

	*Spirea ulmaria*
**Uro1**	*Solidago virgaurea*
	*Capsella bursa pastoris*

These 70% alcohol plant extracts were accurately elaborated by Sevene Pharma according to present medical knowledge.

## Methods

### 1. Chemicals

The R formulation used was Roundup GT+^® ^(Monsanto, Anvers, Belgium) at 450 g/l of G, product number 2020448 available on the market. Dilutions were prepared in Eagle's modified minimum essential medium (EMEM; Abcys, Paris, France), supplemented with 10% calf fetal serum from Cambrex (Verviers, Belgium) or otherwise indicated. G* was radiolabelled by PerkinElmer (Courtaboeuf, France), and has a specific activity of 55 mCi/mmol. Dig1, Dig2, Sp1, Uro1 and Circ1 are mixtures of diluted organic plant extracts (Table 1) obtained by Sevene Pharma (Monoblet, France) from original independent macerates. These were diluted in 70% alcohol. Each solution was prepared in EMEM at 2% of the mixture in positive controls. Bisphenol A (BPA, lot 239658), Atrazine (Az, lot 49085), 3-(4,5-Dimethylthiazol-2-yl)-2,5-diphenyl tetrazolium bromide (MTT) and all other compounds, unless otherwise specified, were from Sigma-Aldrich (Saint Quentin Fallavier, France). BPA and Az were prepared in 0.5% DMSO then diluted in serum-free EMEM and adjusted at pH 7.4. The MTT stock solution at 5 mg/ml in phosphate-buffered saline was diluted 10-fold in serum-free EMEM and filtered through a 0.22 μm filter before each use.

### 2. Cell cultures, Roundup and/or Plant Extract Exposures

The hepatoma cell line HepG2 was provided by ECACC, number 85011430. The cells were isolated from a 15 year-old Caucasian boy. The embryonic kidney 293 cell line (ECACC, number 85120602) was provided by CERDIC (Sophia-Antipolis, France). Cells were grown in flasks of 75 cm^2 ^surface from Dutscher (Brumath, France) in medium (M) containing phenol red-free EMEM with 2 mM glutamine, 1% non-essential amino acid, 100 U/ml of antibiotics (mix of penicillin, streptomycin, kanamycin) and 10% fetal calf serum. For treatments, 50,000 cells were plated per well or in flasks depending on the assay (see below), and grown at 37°C (5% CO_2_, 95% air) over a period of 48 h to 80% confluence in 48-well plates (except for G* treatment, which was conducted with 24-well plates). The cells were then exposed to various concentrations of tested products, the media were changed every 24 hours.

### 3. Cell viability assay

The mitochondrial succinate dehydrogenase activity in cells was measured by the MTT test, based on the cleavage of MTT into blue formazan [30,31], adapted in our group by Auvray et al. [32]. The optical density was measured using a luminometer (Mithras LB 940, Berthold, France) at 570 nm. The toxicity was obtained after R or BPA-Az treatments. The protective actions were evaluated by incubations of plant extracts (X) before toxic treatments, and the curative effects after toxic treatments. Protective efficiencies (from LC50 considered as zero efficiency) were calculated by the formulations at 24 h: 100 - [(100 - XR viability) × 100/(100 - R viability)] and at 48 h: 100 - [(100 - XXR viability) × 100/(100 - R viability)]. Curative effects are calculated for 24 h by the difference RX - RM or RXX - RMM for 48 h, or accordingly up to 96 h.

### 4. Cell entry of ^14^C-Glyphosate (G*)

In order to measure the G entry into cells with or without adjuvants of R, HepG2 confluent cells were exposed to R at 0.01% (24-well plates, 500 μl/well). G* was added in serum-free EMEM at a final concentration of 0.266 μmol/ml for a specific activity of 55 μCi/μmol, corresponding to the quantity of G in 0.01% of R (not significantly toxic in these conditions). Dig1 or Dig2 were added to cells at 2%, proven to be nontoxic, before or after G* and R. Attached cells were then washed 3 times with a PBS solution, lysed by crushing after freezing cycles and the radioactivity was counted with 1.4 ml scintillant liquid (Ultima Gold 6013329 in 6 ml polyethylene tubes in Packard counter 1600LR, USA). The amount of ^14^C-Glyphosate (G*) entered in cells was measured in % by counting the radioactivity in pellets, and taking into account the mortality (20%, as in controls, measured by MTT test) according to the formula [G* in pellets × (120/100)] × 100/G* in supernatants.

### 5. Cytochrome activities

The best protective or curative compound Dig2 (for R or BPA-Az, respectively) was chosen to study cytochrome (CYP) activity. The HepG2 cell line was amplified around 80% confluence and cells were plated at 86 × 10^3 ^cells/cm^2 ^in 3 flasks of 175 cm^2 ^for each point. R (final non toxic concentration 0.0157%) was incubated before or after Dig2 or medium (M) alone, by changing the medium every 24 hours. At the end of the incubation time, cells were collected, counted and stored at -80°C. Then, S9 fractions (membrane and cytosolic enzymes) were prepared for each treatment. The medium was removed, and cells dislodged by treatment with 7 ml of trypsin-EDTA (Lonza, France) and washed (PBS, Eurobio, France) twice by centrifugations (70 *g*, 5 min), at room temperature. Cells were then resuspended in 500 μl of 50 mM phosphate buffer pH 7.5 with 0.25 M sucrose, 1 mM DTT, homogenized and centrifuged at 9,000 *g*, at 4°C for 30 min. The supernatants corresponding to the S9 fractions were collected and frozen at -80°C until further evaluation for enzymatic activities. Protein concentration was determined in each S9 fraction according to the Bicinchoninic Acid Protein Assay (Sigma, France).

The cytochrome P450 CYP1A2 and CYP3A4 activities were quantified by the P450 Glo™ assays (V8771, Batch 271384 and V8801, Batch 277348, respectively; Promega, France). Each 2X Cytochrome P450/KPO_4_/Substrate Reaction mixtures containing the S9 fractions were pre-incubated at 37°C for 10 min in white 96-well plates (655075, Batch 08340329, Dutscher, France). The enzymatic reaction was initiated by adding 25 μl of 2X NADPH regenerating system to each well. The plate was then incubated at 37°C for 20 min for CYP1A2; 30 min for CYP3A4. The reconstituted Luciferin Detection reagent (50 μl) was added before mixing 10 sec and incubating at room temperature for 90 min in order to stabilize the luminescent signal. The luminescence was then read with a luminometer (Veritas Turner Biosystems, France). Three independent experiments were performed from three batches of fraction S9.

### 6. GST activity

The protocol was adapted from Habig et al. [33]. Briefly, 160 μg of the human liver S9 fraction (positive control) or 320 μg of S9 cell fractions were mixed with 10 μl of 100 mM GSH and 990 μl phosphate buffer in duplicate. Reduced L-glutathione (GSH) was dissolved in deionized water; a pH 6.5 buffer was prepared by mixing 0.7 volume of 0.1 M KH_2_PO_4 _and 0.3 volume of 0.1 M Na_2_HPO_4_. The reaction was initiated by 10 μl of 100 mM 1-chloro-2,4-dinitrobenzene (CDNB) substrate. The CDNB was dissolved in 95% ethanol at a concentration of 100 mM. After a 90 sec incubation at 37°C, the optical density was measured at 340 nm every 30 sec for 90 sec with a SmartSpec 3000 Spectrophotometer (Bio-Rad, France). Three independent experiments were carried out using three independent batches of S9 fraction.

### 7. Statistical analysis

The experiments were repeated 3 times in different weeks in triplicate (n = 9) unless otherwise specified. All data are presented as the mean ± standard error (S.E.M.). Statistical differences were determined by an unpaired Student *t*-test using significant levels of p < 0.01 (**) and p < 0.05 (*). For the study of cytochromes and GST activity a Student *t-*test was performed using VisualStat^® ^Professional 2003 (Visualstat Computing, USA). This study made it possible to compare the different treatments.

## Results

**Figure 1:**
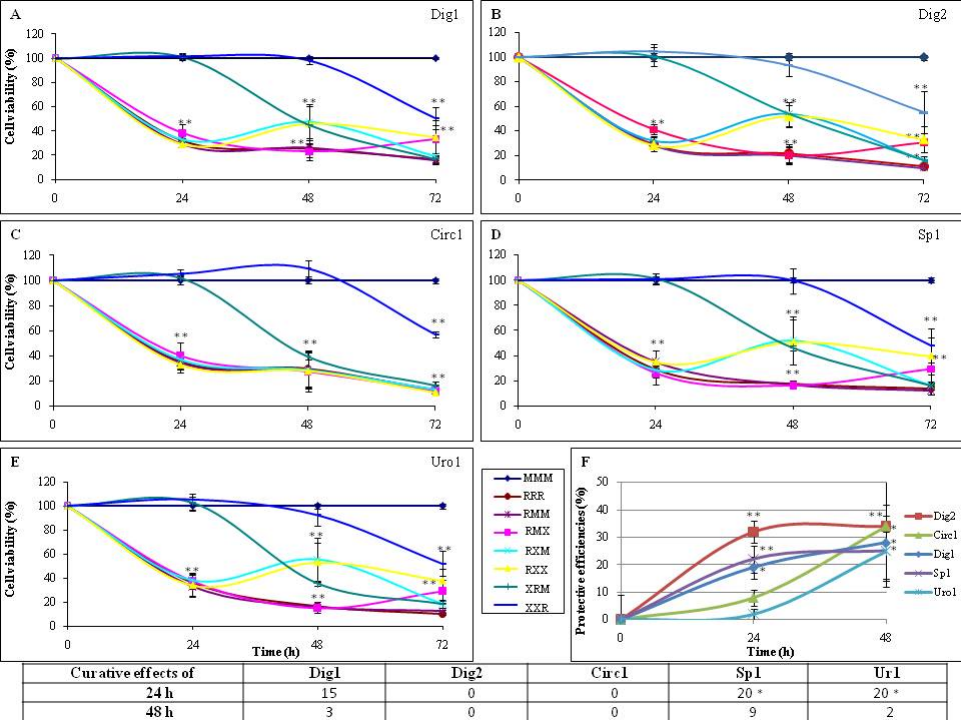
**Preventive and curative effects of various plant extracts on embryonic 293 human cells intoxicated by the herbicide Roundup**. The viability is measured by the mitochondrial succinate dehydrogenase activity in comparison to non-treated cells (M). Cells were grown in EMEM with 10% serum during 48 h, up to 80% confluence in 48-well plates, and then exposed to different treatments. In the frame after A-E, each letter indicates successively 24 h of the following treatments: Medium alone (M) equivalent to Plant extract alone, Roundup alone (R at 0.03%, ~LC50) or with various plant extracts at 2% (X) which are for fig. **A, B, C, D, E**: Dig1, Dig2, Circ1, Sp1, Uro1. In Fig. **F**, the corresponding protective efficiencies (from LC50 considered as zero efficiency) are calculated by the formulations at 24 h: 100 - [(100 - XR viability) × 100/(100 - R viability)] and at 48 h: 100 - [(100 - XXR viability) × 100/(100 - R viability)] and are represented by new colors for each product. In the table below, curative effects are calculated for 24 h by the difference RX - RM or RXX - RMM for 48 h. All experiments were repeated 3 times in triplicates. Statistically significant differences are calculated in comparison to M or to the LC50 for fig. **F**, and by a student *t*-test p < 0.01(**) and p < 0.05(*).

**Figure 2:**
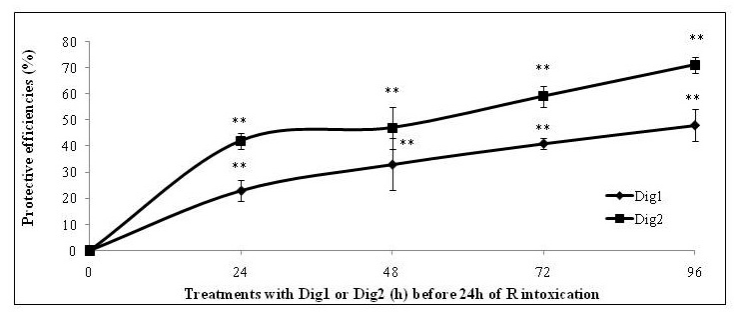
**Protective efficiencies of Dig1 and Dig2 on HepG2 cells intoxicated by Roundup**. These are calculated as indicated in figure 1. Cells were exposed first to Dig1 or Dig2 at 2% during 0-96 h, and then intoxicated 24 h with R (at 0.0175%, ~LC50) or not (control 100%) before this viability measurement.

**Figure 3:**
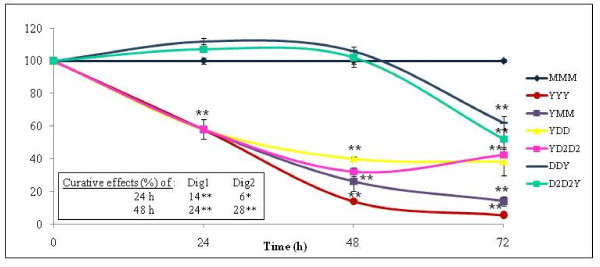
**Preventive and curative effects (% according to time) of Dig1 and Dig2 on HepG2 cells intoxicated by the mixture bisphenol A and Atrazine**. The protocol and calculations are similar to figure 1. In the legend on the right, each letter indicates successively 24 h of the following treatments: Medium alone (M) equivalent to Plant extract alone, mixture of pollutants BPA+Az (called Y at 200 μM each, ~LC50), or with Dig1 (D) or Dig2 (D2) at 2%. In the table in frame, the curative effects are indicated in %.

In total, five plant extracts were studied as medicinal mixtures (Table 1), verified to be nontoxic on the cell lines at 2%. These extracts were then incubated with cells before and after xenobiotic intoxications, either by R (Figure 1, 2) or with the BPA-Az mixture (Figure 3), applied at the related LC50 which was the first one to be determined.

### 1. On human embryonic cells

Figure 1 presents the first screening and characterization of the protective and curative effects (curves and table, respectively) of the 5 plant extracts on embryonic human E293 cells, which were intoxicated by R. It is an efficient toxicant since the corresponding LC50 measured is ~0.03% over only 24 h. This represents our model of intoxication of less differentiated and sensitive cells. Each panel of Figure 1, A to 1E, represents the effect of one plant extract incubated before or after the intoxication by R, according to the time-sequence indicated by different coloured lines in the frame. Dig2 was the most protective, preventing 32-34% of R toxicity in 24-48 h (visible in the upper curve, Figure 1F). Circ1 was comparable but needed a 48 h exposure; most of these effects are dose or time-dependent. Dig1, Sp1 and Uro1 presented a second range protective efficiency reaching 25-28% within 48 h. Curative effects of all these products after R intoxication were more difficult to highlight, and visible essentially after 24 h for Sp1 and Uro1 treatments, with 20% of cells more viable.

### 2. On human HepG2 hepatocytes

Dig1 and Dig2 were chosen as mixtures in order to focus on human HepG2 in Figure 2, because their compounds have active effects on the digestive sphere, according to the bibliography and the medical indications. We first demonstrated again that R was very toxic on such cells, with a LC50 around 0.0175% (175 ppm) over a 24 h period. Dig2 presented again the best protective effects, and Dig1 was representative of the second class, as identified above. We then focused on protective actions of plant extracts over longer periods of up to 96 h, this taking place before the intoxication by R (mixture of G with adjuvants) over a 24 h period. A strong time-dependent hepato-protective effect was then highlighted for both compounds, reaching at least 71% within 96 h for Dig2 or 48% for Dig1.

Then the next goal was to check the specificity of the hepatoprotective effects with another mixture of xenobiotics, equally composed of BPA and Az (Figure 3). The mixture was also demonstrated to be toxic for HepG2 cells with a LC50 of 200 μM for each compound. Surprisingly, both agents had similar properties, no protective effects were highlighted: curves DDY and D2D2Y were equal to Y alone. However, mild time-dependent curative effects (up to 28%) were demonstrated for the first time.

**Figure 4:**
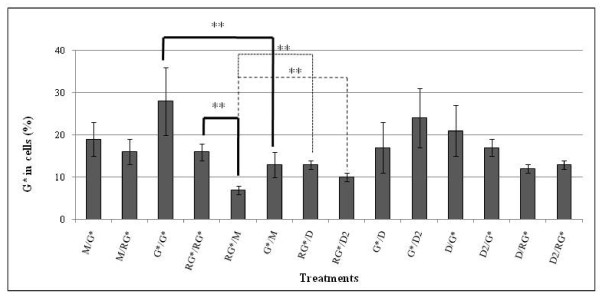
^**14**^**C-glyphosate entered in HepG2 cells in presence or absence of Roundup and Dig1 or Dig2**. Cells were grown in 24-well plates and other conditions and calculations are explained in figure 1. ^14^C-Glyphosate (G*) entry in cells was measured in % by counting the radioactivity in pellets, and taking into account the mortality (20%) according to the formula [G* in pellets × (120/100)] × 100/G* in supernatants. The treatments were changed every 24 h, cells were treated 48 h: for instance, medium (M) for the first day and G* for the second day will be indicated M/G*. G* is at a final concentration of 0.266 μmol/ml for a specific activity of 55 μCi/μmol, corresponding to the quantity of G in 0.01% of R (nontoxic in these conditions). Also, G* was a tracer in a 0.01% dilution of R (RG*), including the adjuvants; the G final concentration was still nontoxic. Dig1 (D) or Dig2 (D2) at 2% or medium alone (M) were incubated during the first or the second day to study preventive or curative effects. The only statistical differences between two treatments are indicated by ** (p < 0.01).

In order to detail the specific mechanisms of protective and curative effects, we tried to find out first whether Dig1 and Dig2 were able to prevent G entry into HepG2 cells (Figure 4). R is composed of G and adjuvants, as already indicated. This was measured by the entry of labelled G (G*) at non toxic doses, as a tracer in presence or absence of these plant extracts. Firstly, we observed that G* (alone or with R adjuvants) entered HepG2 cells in a time-dependent manner (about twice as much in 2 days than in one: G*/G* higher than G*/M, or RG*/RG* higher than RG*/M, statistical differences indicated in bold lines). It was quite clear that the plant extracts could not modify G* penetration by themselves over 24 h, either before or after exposure (no difference between (D or D2)/G* or RG* and M/(G* or RG*); and no difference between G*/M and G*/(D or D2)). However, they apparently lightly promoted G* penetration into cells by ~1.5, but only when R adjuvants were present (statistical comparisons in dotted lines). But R adjuvants did not significantly influence G penetration over 24 h, they could even retain it temporarily, but this effect needs to be confirmed.

**Figure 5:**
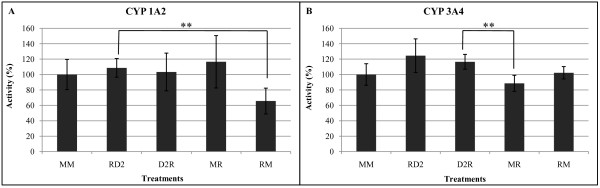
**Effects of Roundup 450 and Dig2 on cytochrome activities in HepG2 cells**. Results are presented in arbitrary units of cytochromes CYP1A2 (5A) and CYP3A4 (5B) activities. Cells were grown in flasks, other conditions and calculations are explained in figure 1. On the abscissa, each letter (M, R, D2) indicates 24 h of successive cell exposures to the corresponding conditions (Medium alone, Roundup, Dig2) and treatments are changed each 24 h. R was applied at 0.0157% (non cytotoxic dose in these conditions) and Dig2 at 2%.

**Figure 6:**
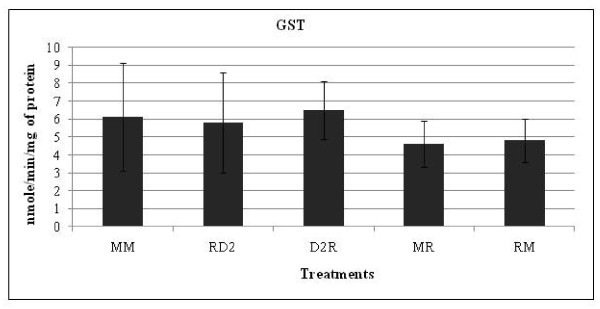
**Effects of Roundup 450 and Dig2 on glutathione S-transferase activity in HepG2 cells**. Cells were grown and treatments performed as explained in figure 5.

As Dig1 and Dig2 do not appear to have developed their protective effects outside cells on G and R entry into cells, we measured Dig2 intracellular actions at three independent endpoints, two major cytochromes P450 induced by xenobiotics, CYP1A2 and CYP3A4 (Figure 5), and GST (Figure 6). We focused on Dig2 since Dig1 had previously been studied, although with another R formulation mixture [9], and because Dig2 presented the most important protective or curative effects depending on the model (Figure 1, 2, 3). There were no major modifications of the cytochromes P450 with the R formulation used in this research, however R appeared to reduce significantly the CYP1A2 activity by 40% (Figure 5A), and this was restored by Dig2 applied after R (curative effect). No preventive effect was highlighted through CYP1A2 regulation, but a mild inhibition (15%) of CYP3A4 was totally restored (Figure 5B). On the other hand, Dig2 did not seem to modulate GST (Figure 6), either in a preventive or curative manner, nor did it modulate the R used in the present study in a significant manner.

## Discussion

First of all, the present work highlighted the toxic effects of an R formulation on human cell lines, whereas R is the most widely used herbicide worldwide. As previously demonstrated, R had cytotoxic effects on embryonic [10] and hepatic cells [8,9]. Here we found that the R formulation used had a LC50 of 0.0175% (175 ppm) for HepG2, ranging between 57-114 times below agricultural levels (1-2%). The LC50 is also ~2.3 times below the maximum level of residues authorized in some genetically modified R-tolerant feed, (400 ppm, [34]). The value of the LC50 demonstrated here is even around 5 times less, if we calculate it from G absolute concentration, and not from a dilution of the commercial product considered as 100%. BPA and Az were used at 200 μM each, which represents approximately the LC50 in short term specific conditions relative to these cells. As this is the case for example with the urine levels of contaminated people (around 16 nM for BPA [35], or 2 nM for Az, [36]. However, such compounds are either lipophilic or have lipophilic residues, which implies that they will bioaccumulate in tissues. Moreover BPA is known to leak from cans (around 2 μM for instance, [37], and 876 μM may form DNA adducts *in vivo *in mice [38]. As far as Az is concerned, it disrupts oocyte maturation at 200 μM [39], and from μM levels, it modifies estrogen synthesis in sensitive target tissues [40]. We also assessed cell viability after BPA-Az intoxication. These products were previously demonstrated as being toxic in HepG2 separately, and in placental JEG3 cells, but they were also capable of disrupting crucial enzymes for cell metabolism or endocrine regulation [6,41,42]. We found that the cell sensitivities depended on the nature of the xenobiotic mixtures and the types of cells used. R was the most toxic of all in these conditions.

Few studies, if any, deal with the prevention and detoxification of contaminants in mixtures taking into account the synergistic effects [6] they may present in the environment. In a similar manner, several plants alone were known for preventive or curative actions, however their synergistic potential was also mostly ignored at a molecular level. Here we highlighted that all the chosen plant mixtures (Circ1, Dig1, Dig2, Sp1, Uro1) may prevent quite effectively embryonic cell mortality up to 1/3 to 1/4 within 1-2 days only, if administered before the intoxication; although with different kinetics demonstrating cellular specific effects. The anti-pollutant effect is greater during prevention than if the plants are administered after toxicants (1/5 of recovery only with two compounds, Sp1 and Uro1), probably because the lethal effects of R are amplified with time [10]. *Sambucus nigra *in Sp1 or *Solidago virgaurea *in Uro1 are known for their protective cell actions, overall against oxidative molecules [22,43,44] that could be present in R formulation. Similarly, the plant protective properties are also time-dependant. The specificity of action was better confirmed when the protection efficiency reached up to 71% with hepatoprotective agent Dig2 applied on hepatocyte-derived cells. This was true even if HepG2 cells were about 1.7 more sensitive to intoxication than embryonic cells in this case (from LC50 comparisons for R). We also deduced the specificity of plant mixtures actions thanks to the fact that the effects were different with another type of pollutants, for instance, the curative effects reached 28% for Dig 2 after HepG2 intoxication by BPA-Az. Therefore, it seems that oxidative damage protection by plant extracts cannot explain all the effects. Moreover, with R tested on other human cells, we know that numerous enzymes were reached, such as adenylate kinase, caspases 3/7 [7], aromatase, and even steroid receptors [8].

To approach the molecular actions of the most hepatoprotective compounds, we tested the importance of an extracellular trap of contaminants using the plant extracts. We also made this assumption because the plant extracts did not have any cytotoxicity by themselves in our conditions. A major extracellular trapping was almost excluded by the fact that a labelled contaminant was penetrating the cells with or without the R formulation. The plant extracts together with the R adjuvants might have even helped G penetration. It was deduced that the effects were mostly intracellular. Some G metabolites may even have been already excreted, and this does not exclude a bioaccumulation of contaminants over longer periods of time.

Dig2 had the major protective and curative effects on HepG2; it contains *Chelidonium majus*, which is also known to reduce transaminase-enhanced levels by toxicants *in vivo *[45,46]. Moreover, Dig2 also contains extracts of *Raphanus sativus*, which is mainly composed of glucosinolates. These are transformed in part into indole-3-carbinol, which in turn induces enzymes of hepatic phase I metabolism of xenobiotics, such as cytochrome P450 [47]. This may contribute to the elimination of toxicants and could therefore prevent their adverse effects, at least in part. Several studies have indicated that HepG2 cells retained the activities of the drug metabolism phase I and phase II enzymes involved in activation and detoxification of genotoxic carcinogens [48,49]. As a matter of fact, Dig2 restored CYP1A2 or prevented CYP3A4 depression, following R intoxication in the present work. Other cytochromes like human hepatic CYP17 are also inhibited by pollutants such as BPA and nonylphenol [50,51]. Altogether this shed a new light on the intracellular actions of these plant extracts but other enzymatic impacts cannot be excluded. We thus measured GST in HepG2, but its absence of modulation by Dig2 or R underlines again the specificity of intoxication/detoxification pathways.

In conclusion, we demonstrated specific and cytotoxic effects of R and BPA-Az on human hepatocytes-derived and embryonic cell lines. This was for R at doses far below those used in agriculture and at levels of residues present in some genetically modified food and feed. In these conditions, cell mortality induced by R can be almost entirely prevented in HepG2 cells within 48 h by Sevene Pharma products Dig1 and Dig2. The latter also had some curative effects after BPA-Az intoxication. Similarly Circ1, Sp1 and Uro1, had some protective or curative effects depending on the cells and the toxicants. The pathways involved comprise at least CYP1A2 and CYP3A4 after R and Dig2 actions, thus the defense systems of the cells are modulated. Besides promising actions that need to be confirmed *in vivo*, these products provide altogether a useful tool to better understand the intoxication/detoxification pathways reacting in case of physiological contamination by xenobiotics.

## Competing interests

The authors declare that they have no competing interests. The developments of plant extracts in Sevene Pharma were performed completely independently of their biological assessments. The scientists in the University of Caen in charge of the assessment of xenobiotics or plant extracts declare no financial or other interests in the development of these products.

## Authors' contributions

CG carried out the cellular, biochemical and molecular studies, participated in drafting the manuscript. CL participated in plant extracts conception and discussions. CDL directed formulations and assessments of medicinal plant extracts Circ1, Dig1, Dig2, Sp1 and Uro1 for Sevene Pharma. RM and EC reproduced and helped the cellular experiments. CT participated in the methodological and protocol advices, and discussions. GES conceived the study, the final version of the manuscript, was responsible for the design of the work and was the scientific head and coordinator. All authors read and approved the final manuscript.
